# Comparison between refractive outcomes of femtosecond laser-assisted cataract surgery and standard phacoemulsification

**DOI:** 10.1186/s12886-019-1277-9

**Published:** 2020-01-02

**Authors:** Mohamed Shafik Shaheen, Amir AbouSamra, Hany Ahmed Helaly, Amr Said, Ahmed Elmassry

**Affiliations:** Ophthalmology Department, Faculty of Medicine, Alexandria University, 30 Roshdystreet, Roshdy, Alexandria, Egypt

**Keywords:** FLACS, Femtosecond, Cataract, Phacoemulsification, Refractive outcomes

## Abstract

**Background:**

To compare the visual and refractive outcomes of femtosecond laser assisted cataract surgery (FLACS) using Victus platform (Technolas Bausch and Lomb (B&L), Munich, Germany) and conventional phacoemulsification cataract surgery (CPCS).

**Methods:**

A retrospective study of 100 eyes operated for cataract. FLACS was performed in 50 eyes and CPCS was done in another 50 eyes. Preoperative and 6 months postoperative visual and refractive evaluation (efficacy, safety, predictability, and surgically induced astigmatism) as well as higher-order aberrations were analyzed. Efficacy index which equals post-operative mean of uncorrected distance visual acuity (UDVA) divided by preoperative mean corrected distance visual acuity (CDVA) was calculated in both groups. Safety index equals post-operative mean of corrected distance visual acuity (CDVA) divided by preoperative mean CDVA.

**Results:**

Logarithm of the Minimum Angle of Resolution (LogMAR), UDVA improved in both groups after surgery (*p* < 0.05). It was 0.23 ± 0.20 and 0.291 ± 0.311 log MAR in FLACS and CPCS groups respectively. Safety index was 1.777 in FLACS group and 1.744 in CPCS groups showing high degree of safety of both measures. Mean surgically induced astigmatism (SIA) was 0.35 ± 0.67 D and 0.901 ± 0.882 D in FLACS and CPCS groups respectively (*p* = 0.015). The post-operative comparison between both groups was in favor of CPCS group vs. FLACS group regarding total aberrations (0.563 ± 0.386 vs. 0.91 ± 1.20) (*p* = 0.03), while low order aberrations were significantly less in FLACS group vs. CPCS group (0.64 + 0.63 vs. 2.07 + 3.15) (*p* = 0.027). RMS high order aberration was higher in FLACS group vs. CPCS group but of no statistical significance 0.54 ± 0.96 vs. 0.328 ± 0.360 (*p* = 0.082).

**Conclusion:**

Femtosecond laser -assisted cataract surgery was a safe and precise procedure but enhanced visual outcomes only minimally when compared to conventional cataract surgery in experienced hands. Both FLACS and manual surgeries can achieve a high efficacy, predictability and safety with slight superior outcomes in FLACS.

**Trial registration number:**

PACTR201804003256258 (date: 27 Mar 2018) Available at: https://pactr.samrc.ac.za/

## Background

The great work of Charles Kelman has introduced a revolutionary shift from extra capsular cataract extraction surgeries to phacoemulsification procedure [1]. Since then, several trials have been exerted to provide a higher safety and efficacy levels in cataract surgeries. The introduction of premium intraocular lenses (IOLs) as toric IOLs for astigmatism correction or multifocal IOLs for restoring near vision raised surgeon concerns of creating highly accurate reproducible capsulotomies and more precise self-sealed corneal incisions to achieve the highest possible degree of postoperative spectacle independence [1]. Femtosecond laser technology was believed to provide a further step in this issue [2].

Several modifications in platforms and software updates have been achieved since Nagy ZZ performed his first femtosecond laser-assisted cataract surgery (FLACS) about 10 years ago [3]. It is widely accepted now that FLACS yield a high degree of accuracy and reproducibility of capsulotomies size regularity [4] with less effective phaco-time [5]. However, the clinical impact of these advantages is still under investigations.

Most published study results ensure that FLACS is better or at least equal to conventional phacoemulsification cataract surgery (CPCS) regarding safety and efficacy [6, 7]. The issue of being cost-effective has been strongly investigated without a final decision till now [8, 9]. The main concern of most cataract surgeons now regarding FLACS is whether the high degree of precision and circularity of capsulotomy will yield a better visual and refractive outcome enough to make FLACS cost effective [10]. The proposed refractive superiority of FLACS is believed to result from various factors; high precision and reproducibility of both corneal incisions and capsulotomies size and shape are the main factors. Precision of corneal incisions yields less surgically induced astigmatism [11].Accuracy of capsulotomy produces more intraocular lens (IOL) centration with minimal decentration and tilt that have unpleasant refractive effect on both short-term and long-term outcomes [3].

A strong potential of FLACS is the ability to correct mild to moderate degrees of astigmatism during the same surgical procedure at no extra cost or time. Highly accurate laser astigmatic keratotomies can be placed on the cornea either as penetrating incisions or intrastromal incisions correcting up to 2.5 diopters (D) of astigmatism with some reports of efficacy equal to toric IOLs in this astigmatism range [12]. This ability can be used to achieve high degrees of postoperative spectacle independence which is a major concern of recent advanced cataract refractive surgeries techniques**.**

The purpose of this study was to compare the visual and refractive outcomes of femtosecond laser assisted cataract surgery using Victus platform (Technolas B&L, Munich, Germany) and conventional phacoemulsification cataract surgery (CPCS). This study, to our knowledge, is the first large Egyptian database discussing refractive outcomes and assessing abberations in FLACS compared to conventional surgery. The Victus is a relatively newer platform in the Egyptian market and has not been evaluated well.

## Methods

This was a retrospective interventional clinical study comparing femtosecond laser-assisted cataract surgery cases (study group) and conventional phacoemulsification cataract surgery cases (control group). The study included 100 eyes that were divided into two groups. The eyes included were operated upon consecutively during the period of July 2017 to July 2018. No cases were excluded due to intraoperative complications e.g. posterior capsule rupture. The first group included 50 eyes of 50 patients who had undergone FLACS. The second group included matched 50 eyes of 50 patients for whom CPCS was done. The selection of either procedures was based upon patient’s choice according to his affordability of FLACS. The included cataract patients had an age range of 18–80 years having visually significant cataract. Cases of glaucoma, corneal opacity, macular diseases, and any other problem expected to affect vision were excluded from the study. Cataract grading was done using LOCS III classification [13]. All cases were operated by the same surgeon Ahmed El Massry (A.M.). All cases had a single piece monofocal hydrophobic acrylic aspheric IOL implantation (Tecnis-1; AMO, advanced medical optics, USA) with lens power chosen according to the results of optical biometry, (IOLMaster, Carl Zeiss Meditec or Lenstar LS 900, Haag-Streit).

The study protocol was approved by the local Ethical Committee of the Faculty of Medicine, Alexandria University, Egypt. Tenets of Declaration of Helsinki were followed. An informed written consent was obtained from all patients to whom all the details of the procedure were explained with emphasis on the intended outcome and possible complications**.**

Preoperative evaluation was conducted to all patients including: complete anterior segment examination with slit-lamp biomicroscopy, uncorrected distance visual acuity (UDVA), manifest and cycloplegic refraction, corrected distance visual acuity (CDVA), applanation tonometry, fundus examination, corneal specular microscopy and topography as well as I-trace ray tracing aberrometer (Tracey Technologies, Houston, USA)**.**

### Surgical technique

Preoperatively on the day of surgery, all patients received topical anesthesia with benoxinate 0.4% eye drops and their pupils were dilated using phenylephrine 2.5%, cyclopentolate 1.0% and /or tropicamide 1.0% eye drops.

### Femtosecond laser-assisted cataract surgery

Using the Victus femtosecond laser system (Baush&Lomb, Technolas Munich, Germany) in the study group, corneal incisions, the main wound (2.4 mm) and two side-ports (1.2 mm) were created. Capsulotomy and lens fragmentation were performed also using the femtosecond laser. Real time high-resolution video and anterior segment spectral-domain OCT imaging was used to plan and monitor treatment especially for detection of posterior capsule and iris margin safety zones. After the laser procedure, the patient was then transferred to the operating room for completion of surgery. Corneal incisions were opened using a flap lifter and the anterior chamber was filled with viscoelastic. Then, the anterior capsule was removed using a capsulorhexis forceps following the contour of the laser capsulotomy. Careful hydrodissection was done avoiding exertion of excessive pressure through the cannula (to prevent capsule block). Surgery was then completed using standard phacoemulsification and the procedure was followed by intraocular lens implantation in the capsular bag after removal of the lens cortex.

### Conventional phacoemulsification cataract surgery

Corneal incisions were created using a 2.4 mm keratome for main incision placed in all cases on the steeper topographic axis as determined using corneal topography and two 1.2 mm incisions were obtained using a microblade for the side ports. The anterior chamber was filled with viscoelastic. The continuous curvilinear capsulorhexis (CCC) was created with a capsulorhexis forceps. Lens segmentation was performed using a divide-and-conquer approach. The procedure was followed by intraocular lens implantation in the capsular bag after removal of the lens cortex.

For both groups; hourly topical antibiotics and steroids eye drops were used for 24 h then every 4 h for 1 week. This was followed by gradual tapering of steroids over 2 weeks. Postoperative follow-up for all patients was done at 1and 6 months period where UDVA, CDVA, and manifest refraction measurements were obtained. Wave-front errors (high order aberrations; coma, trefoil, and spherical aberration) were measured at the 6th month using I-trace ray tracing aberrometer (Tracey Technologies, Houston, USA).

### Main outcome criteria

Uncorrected distance visual acuity (UDVA) and corrected distance visual acuity (CDVA) were measured and expressed in logMAR units for statistical analysis at 1 month and 6 months following the cataract surgery. Rules mentioned by Holladay JT [14] for calculating the average visual acuity were followed. Refractive error (spherical as well as astigmatic refractive errors in both magnitude and axis) was obtained at 1 and 6 months postoperatively. According to the postoperative intended refraction, the eyes were classified into 4 groups. Percentages of eyes within 0.25 diopter, 0.5 diopter, 1 diopter, and > 1 diopter of intended refraction at the 6th postoperative month were reported. Surgically induced astigmatism was calculated for all cases. Root mean square (RMS) total eye aberrations, lower order and higher order aberrations (coma, trefoil, and spherical aberrations) were measured using the I-Trace ray tracing aberrometer at 6 months following the surgery. Measurements were obtained without pupillary dilatation in a semi dark room.

### Vector analysis for astigmatism

Preoperative and 6 months postoperative values for total astigmatism were analyzed using the Alpins method [15] in which the preoperative and post-operative K-readings and their axes were used to evaluate the effective change in astigmatism value with consideration of the change in the astigmatic axis.

The target-induced astigmatism (TIA) is the astigmatic change (magnitude and axis) that the surgery was planned to induce. The surgically induced astigmatism (SIA) is defined as the amount and axis of the astigmatism that was induced by the surgery. The correction index is calculated as a ratio between the SIA and the TIA. Perfectly, it should equals 1.0; with values > 1.0 considered an overcorrection and < 1.0 indicating undercorrection.

Data analysis was performed using the software SPSS for Windows version 20.0 (SPSS Inc., Chicago, USA). Quantitative data were described using range, mean and standard deviation. Normality of data samples was evaluated using the Kolmogorov-Smirnov test. Paired t test was used for comparisons between means of the preoperative and postoperative data. Chi-square test was used to compare between different percentages. Pearson Correlation coefficient was used to assess the correlation between different variables. Standard Figures for reporting the outcomes in refractive surgery, according to the Waring Protocol and its modification, were used for displaying and summarizing the refractive outcomes of this study for each group postoperatively [16, 17].

## Results

This study included 100 eyes having visually significant cataract that have been operated using either FLACS (50 eyes) or CPCS (50 eyes). Table 1 shows preoperative demographic, visual and refractive data of patients in both groups. There was no statistically significant difference between both groups.

**Table 1:** Preoperative demographic, visual, and refractive data of patients in both groups

	Group 1 “FLACS”		Group 2 “CPCS”		*P* value
Age (years):					
Range	(45–80)		(55–77)		0.379
Mean ± SD	66.52 ± 8.91		66.04 ± 2.53		
Side:					
right	23	(46%)	26	(52%)	0.248
left	27	(54%)	24	(48%)	
Sex:					
Male	25	(50%)	23	(46%)	0.50
Female	25	(50%)	27	(54%)	
Cataract grade:					
Range	(1–4)		(1–4)		
Mean ± SD	2.40 ± 0.90		2.38 ± 0.82		0.23
IOP (mmHg):					
Range	(10–21)		(10–20)		0.430
Mean ± SD	13.22 ± 2.35		13.10 ± 4.13		
AL (mm):					
Range	(21.65–31.3)		(21.7–31.0)		0.13
Mean ± SD	24.36 ± 2.44		24.16 ± 2.06		
IOL (diopter):					
Range	(−2–28)		(− 2–25)		0.184
Mean ± SD	18.48 ± 7.56		19.80 ± 6.70		

*FLACS* Femtosecond laser assisted cataract surgery

*CPCS* Conventional phacoemulsification cataract surgery

*IOP* Intraocular pressure

*AL* Axial length

*IOL* Intraocular lens

**Fig. 1:**
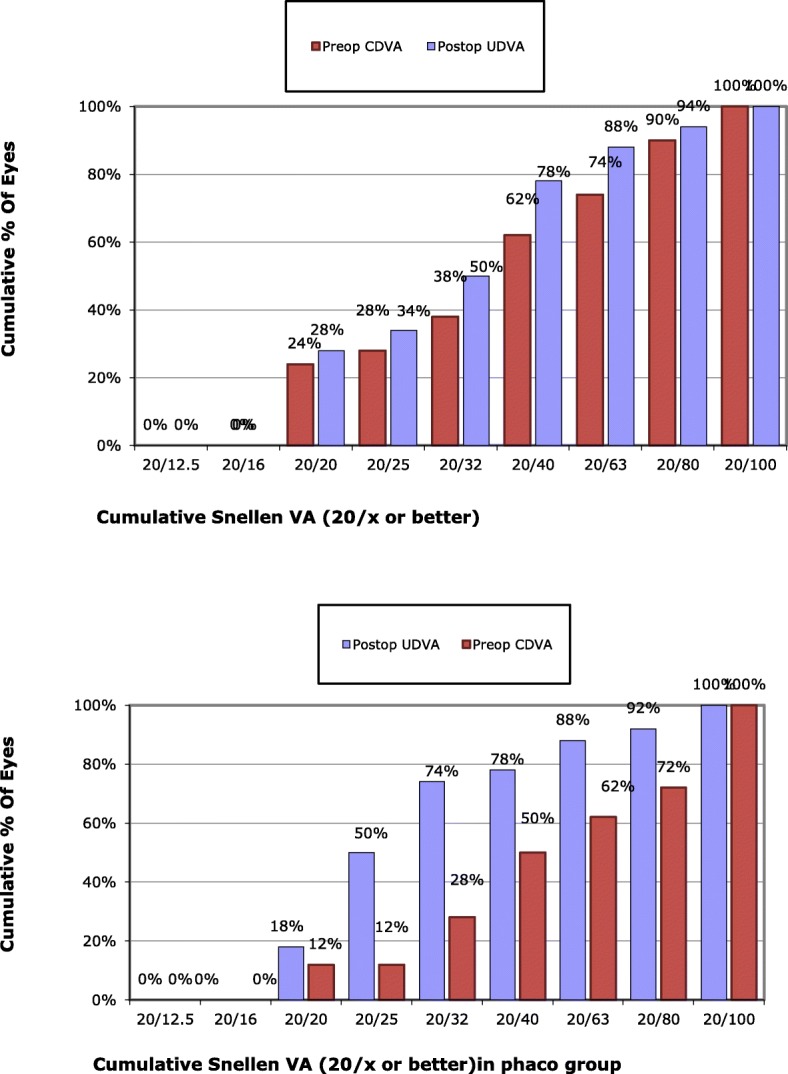
Cumulative percent of eyes with various Snellen’s visual acuity in femtosecond group (above) and phaco group (below)

**Fig. 2:**
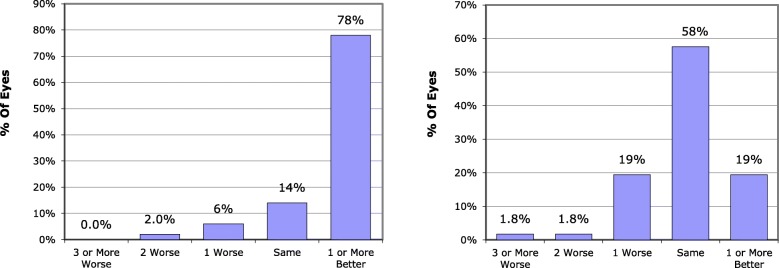
Differences between postoperative uncorrected distance visual acuity and preoperative corrected distance visual acuity in femtosecond group (left) and phaco group (right)

Table 2 and Figs. 1 and 2 show the visual outcome of surgery in eyes undergoing FLACS and CPCS. LogMAR UDVA improved in both groups after surgery (*p* = 0.001). Following surgery, logMAR UDVA and CDVA were slightly better in the FLACS group but the difference was not statistically significant. Postoperative improvement of logMAR CDVA in both groups was statistically significant (p = 0.001). Efficacy index which equals post-operative UDVA divided by pre-operative CDVA was calculated in both groups revealing an efficacy of 1.266 and 1.418 in FLACS and CPCS groups, respectively. Safety index equals post-operative mean CDVA divided by preoperative mean CDVA. This index was 1.777 in FLACS group and 1.744 in CPCS groups showing high degree of safety of both measures. Regarding uncorrected and corrected near visual acuities, similar results were obtained for both groups. Details were not mentioned to avoid redundancy in the manuscript. There was a statistically significant improvement postoperatively from preoperative levels.

**Table 2:** The visual outcome of surgery in eyes undergoing femtosecond laser-assisted cataract extraction and conventional phacoemulsification cataract surgeries

	Group 1 “FLACS”	Group 2 “CPCS”	*P* value
Preop. UDVA (logMAR):			
Range	(0.0969–1.778)	(0.301–1.301)	0.007^a^
Mean ± SD	0.89 ± 0.44	0.64 ± 0.39	
Postop. UDVA (logMAR)			
Range	(0–1.301)	(0–1.301)	0.116
Mean ± SD	0.23 ± 0.20	0.291 ± 0.311	
P_2_ value	0.001^a^	0.001^a^	
Preop. CDVA (logMAR):			
Range	(0–1.778)	(0–1.301)	0.326
Mean ± SD	0.50 ± 0.47	0.54 ± 0.45	
Postop. CDVA (logMAR)			
Range	(0–0.301)	(0–0.301)	0.069
Mean ± SD	0.10 ± 0.10	0.136 ± 0.123	
P_2_ value	0.001^a^	0.001^a^	

*UDVA* Uncorrected distance visual acuity

*CDVA* Corrected distance visual acuity

^a^statistically significant

Postoperative intraocular levels were not statistically significant from preoperative levels in both groups (*p* > 0.05). In CPCS group, the mean intraocular level was 13.11 + 2.33 mmHg and 13.21 + 2.41 mmHg at 1 and 6 months following the surgery respectively. In FLACS group, the mean intraocular level was 13.08 + 2.13 mmHg and 13.11 + 2.31 mmHg at 1 and 6 months post-operative respectively.

Table 3 shows the refractive outcome of surgery in eyes undergoing FLACS and CPCS. Improvement of refractive error was encountered in both groups following surgery (*p* = 0.0149, 0.0253 respectively). FLACS group patients were more towards myopic side before the surgery; having higher degree of myopic spherical equivalent error and slightly more astigmatism. Those preoperative refractive characteristics have no statistical significance. All refractive parameters showed statistically significant improvement following surgery in both groups.

**Table 3:** The refractive outcome of surgery in eyes undergoing femtosecond laser-assisted cataract extraction and conventional phacoemulsification cataract surgeries

	Group 1 “FLACS”	Group 2 “CPCS”	*P* value
Preop. Spherical error (D)			
Range	−20 - 3.75))	− 20 – 3))	0.088
Mean ± SD	− 2.22 ± 5.72	−1.76 ± 4.52	
Postop. Spherical error (D)			
Range	(−2–1.25)	(−3–1.25)	0.073
Mean ± SD	− 0.15 ± 0.76	− 0.372 ± 0.913	
p_2_ value	0.0149^a^	0.0253^a^	
Preop. MRSE (D)			
Range	(− 21–3.25)	(− 20.1–2.625)	0.086
Mean ± SD	−2.74 ± 5.87	−2.16 ± 4.54	
Postop. MRSE (D)			
Range	(−2.5–1)	)-3.625–1)	0.049^a^
Mean ± SD	− 0.48 ± 0.80	− 0.765 ± 0.908	
p_2_ value	0.0043^a^	0.0206^a^	
Preop. Total astigmatism (D)			
Range	)-2–0)	)-2–0)	0.381
Mean ± SD	− 1.06 ± 1.02	− 1.00 ± 0.55	
Postop. Total astigmatism (D)			
Range	)-2–0.75)	)-2–0)	0.162
Mean ± SD	− 0.64 ± 0.62	−0.786 ± 0.530	
p_2_ value	0.0021^a^	0.023^a^	
Preop. Corneal astigmatism (D)			
Range	)-2–0)	(−2–0)	0.113
Mean ± SD	−1.02 ± 0.95	−0.97 ± 0.49	
Postop. Corneal astigmatism (D)			
Range	(0 – −1.75)	(0 - -2)	0.248
Mean ± SD	−0.66 ± 0.73	−0.776 ± 0.543	
p_2_ value	0.0004^a^	0.001^a^	

*MRSE* Manifest refraction spherical equivalent, ^a^statistically significant

Regarding refractive predictability, the achievement of target manifest refraction was measured by calculating the absolute difference between target refraction and the post-operative spherical equivalent. The mean absolute error was 0.56 ± 0.50 D (range 0-2D) and 0.694 ± 0.730 D (range 0–3.2 D) in FLACS and CPCS groups respectively (*p* = 0.178). Mean arithmetic error (measured by calculating the difference between target refraction and the post-operative spherical equivalent) was - 0.11 ± 0.71 D (range − 2 - 1.11 D) and- 0.418 ± 0.932 D (range − 3.2–1.375 D) in FLACS and CPCS groups respectively (*p* = 0.136).

**Fig. 3:**
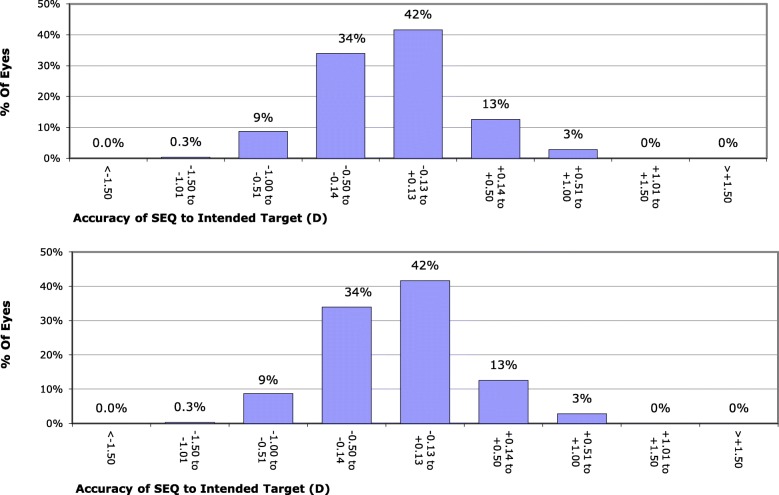
Accuracy of spherical equivalent (SEQ) to intended target in femtosecond group (above) versus phaco group (below)

In the FLACS group, the number of eyes within 0.25 D,0.5 D, 1 D and 1.5 D from target refraction were 34 (68%), 38 (76%).44 (88%), and 48 (96%)eyes, respectively (Fig. 3). While in CPCS group, the number of eyes within 0.25 D,0.5 D, 1 D and 1.5 D from target refraction were 20 (40%),24 (48%), 36 (72%) and 42 (84%)eyes, respectively (Fig. 3). Those values showed a statistically significant difference between the 2 groups (*p* = 0.026, 0.011, 0.032, 0.041, respectively). The accuracy index is defined as the percentage of eyes within 0.5 D of emmetropia. It was calculated for both groups and showed superior predictability of FLACS having 76.0% of eyes in this category in comparison to 48.0% in CPCS phaco group (*p* = 0.011).

Correlation analysis was done to investigate the presence of correlation between mean absolute error (MAE) and patient age, preoperative spherical equivalent (SE), and axial length (AL) in both groups. No correlation was found between MAE and AL in FLACS group (*p* = 0.775). In CPCS group, there was a weak but significant correlation (r = − 0.307, *p* = 0.04), showing higher errors in eyes with long axial length. No correlation was found in both groups between MAE and age or preoperative SE.

**Fig. 4:**
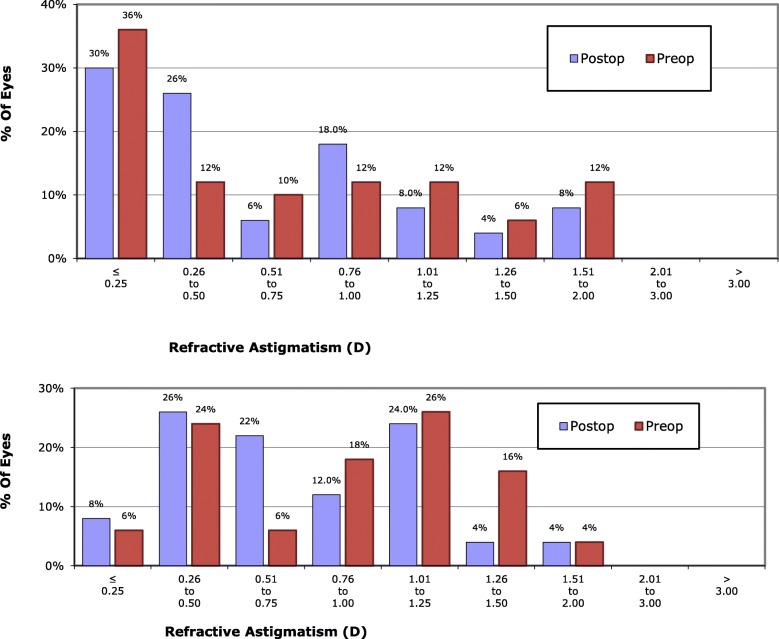
Percentage of eyes at different degrees of astigmatism in femtosecond group (above) and phaco group (below) before and after surgery

**Fig. 5:**
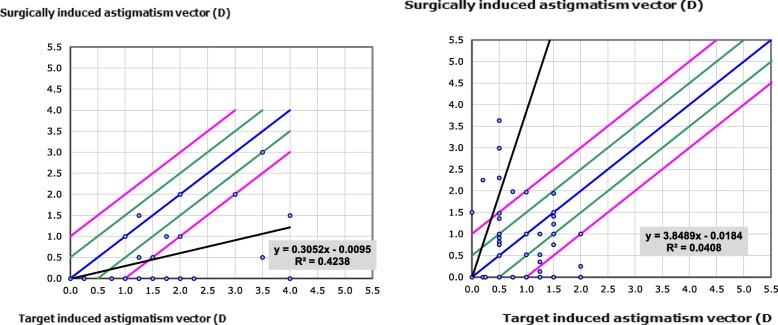
Target Induced Astigmatism versus Surgically Induced Astigmatism in femtosecond group (left) and phaco group (right)

The FLACS group had slightly more astigmatism, both total refractive astigmatism and cornel astigmatism (*p* = 0.381 and 0.113). Total refractive astigmatism had a statistically significant reduction following surgery in both FLACS and CPCS groups (*p* = 0.0021 and 0.023). Corneal astigmatism had a statistically significant reduction following surgery in FLACS and CPCS groups (*p* = 0.0004 and 0.001). Less post-operative astigmatism was noticed in the FLACS group but the difference between both groups was not statistically significant, *p* = 0.248 (Table 3) (Figs. 4 and 5).

Surgically induced astigmatism (SIA) was calculated using vector analysis according to Alpins method from pre- and post-operative topography. Mean SIA was 0.35 ± 0.67 D and 0.901 ± 0.882 D in FLACS and CPCS groups, respectively. It was significantly lower in FLACS group (*p* = 0.015). The correction index (CI) is calculated as the ration of SIA to TIA. The CI value indicates an overcorrection if it is more than 1 or an undercorrection if it is less than 1. CI at 6 months was 0.86 vs. 0.52 for FLACS and CPCS groups respectively. At the end of 6th month, 80% vs. 60% of the eyes were within 15 degrees of the preoperative meridian of astigmatism in FLACS and CPCS groups respectively. Mean angle error was3.9 ± 9.67 degrees and 7 ± 0.87 degrees in both groups, respectively. The angle of error (AE), was very close to 0, indicating no significant systematic error of misaligned treatment.

I-trace was used to assess ocular aberrations before and 6 months following surgery. Data obtained was total RMS, low order (LO) aberrations, high order (HO) aberrations, as well as coma, trefoil, and spherical aberrations (Tables 4 and 5). All mentioned measures improved after FLACS with statistically significant difference in total aberrations (*p* = 0.001), low order aberrations (*p* = 0.0005) and coma (*p* = 0.0023). Change in high order aberrations, trefoil and spherical aberrations was not statistically significant (*p* = 0.2725, 0.2302, 0.080 respectively). The preoperative difference between both groups regarding total, high order and low order aberrations was not statistically significance with slightly higher root mean square total aberrations in FLACS group (mean of 2.23 ± 2.02) than in CPCS group (mean of 2.13 ± 2.48). Less high order aberration was found in FLACS group than in CPCS group preoperatively (mean of RMS equals 0.67 ± 1.27 and 0.84 ± 1.31 in both groups respectively). On the other hand, low order aberrations were more in FLACS group with mean RMS 2.14 ± 3.98 vs. 2.07 ± 3.15 for CPCS group. Again, the preoperative difference represented no statistical significance. Postoperatively, a statistically significant improvement has been achieved regarding RMS total and high order aberration in CPCS group (RMS 0.563 ± 0.386 and 0.328 ± 0.360 respectively). While in FLACS group, a statistical significant improvement has been achieved regarding RMS total and low order aberration (0.91 ± 1.20 and 0.54 ± 0.96), the post-operative comparison between both groups was in favor of CPCS group regarding total aberrations (*p* = 0.03) while low order aberrations were significantly less in FLACS group (*p* = 0.027). RMS high order aberration was higher in FLACS group but of no statistical significance (*p* = 0.082).

**Table 4:** High and low order aberrations in both groups

	Group 1 “FLACS”	Group 2 “CPCS”	*P* value
RMS total			
Pre operative			
Range	(0.22–8.455)	)0.166–8.55)	0.495
Mean ± SD	2.23 ± 2.02	2.13 ± 2.48	
Post operative			
Range	(0.1593–8.99)	(0.11–2)	0.030*
Mean ± SD	0.91 ± 1.20	0.563 ± 0.386	
P2 value	0.001*	0.0001*	
High Order (HO)			
Pre operative			
Range	0.078–8.444))	(−0.7206–5.052)	0.251
Mean ± SD	0.67 ± 1.27	0.84 ± 1.31	
Post operative			
Range	(0.05–6.86)	)0.031–0.84)	0.082
Mean ± SD	0.54 ± 0.96	0.328 ± 0.360	
P2 value	0.2725	0.0034*	
Low Order (LO)			
Pre operative			
Range	0.004–14.063))	(0.129–14.063)	0.422
Mean ± SD	2.07 ± 3.15	2.14 ± 3.98	
Post operative			
Range	(0.08–1.02)	(0.1447–4.49)	0.027*
Mean ± SD	0.64 ± 0.63	2.07 ± 3.15	
P2 value	0.0005*	0.1600

**Table 5:** Coma, spherical aberration, and trefoil in both groups

	Group 1 “FLACS”	Group 2 “CPCS”	*P* value
Coma			
Pre operative			
Range	)-0.043–0.977)	)-0.174–1.5)	0.202
Mean ± SD	0.20 ± 0.20	0.23 ± 0.28	
Post operative			
Range	(0.004–0.811)	(0.016–0.39)	0.371
Mean ± SD	0.10 ± 0.13	0.090 ± 0.089	
p_2_ value	0.0023*	0.0004*	
Spherical aberration (SA)			
Pre operative			
Range	)-0.174–0.475)	(−0.788–0.475)	0.281
Mean ± SD	0.06 ± 0.13	0.08 ± 0.17	
Post operative			
Range	)-0.025–0.245)	(0.003–0.022)	0.009*
Mean ± SD	0.03 ± 0.05	0.011 ± 0.006	
p_2_ value	0.080	0.005*	
Trefoil			
Pre operative			
Range	)0.011–1.4)	(0.06–1.4)	0.431
Mean ± SD	0.19 ± 0.22	0.19 ± 0.24	
Post operative			
Range	)0.001–0.977)	(0.003–0.031)	0.001*
Mean ± SD	0.17 ± 0.23	0.027 ± 0.006	
p_2_ value	0.2302	0.001*	

## Discussion

The introduction of FLACS technology in ophthalmic market has created a strong debate of actual benefit of applying this advanced technology as a part of premium package for refractive cataract patients. FLACS is a modern surgical technique that tries to gain the benefit of femtosecond laser precision in the creation of corneal wounds, anterior capsulotomy and nuclear fragmentation in phacoemulsification surgery. It is generally accepted that the corneal wounds and capsulotomy created by femtosecond laser have a superior precision and reproducibility that is unmatched by manual methods. The current study tries to assess the impact of this proposed precision on the final visual acuity and refractive outcome as well as changes achieved in both high and low order aberrations.

This superior visual and refractive outcome of FLACS is still until now a hypothesis rather than a true fact. The support of this hypothesis is that FLACS is associated with improved prediction of surgically-induced corneal astigmatism (SIA) and intraocular lens placement. Most existing studies show either no or little improvement in post-operative refraction. Taking the expenses and logistical issues into consideration, comparison between the refractive outcomes of both techniques is a current issue of intense controversy in ophthalmology society.

Both groups achieved a high degree of post-operative spherical equivalent refractive predictability. In the femtosecond group, 68% of patients achieved ≤0.25 D of absolute refractive error compared to 40% in the manual group (*p* = 0.026). Lawless et al. [18] found no significant difference in a retrospective consecutive cohort study of 61 eyes that had FLACS and 29 eyes that had manual phacoemulsification. In a comparison of 48 eyes operated on with femtosecond laser technology and 51 eyes operated on manually, these results were supported by similar findings of Miháltz et al. [19]

Most of studies published in the literature comparing refractive outcomes of FLACS to manual surgery found no statistically significant difference between the surgical methods while some studies found a small statistically significant rather than a clinically significant difference. The sample size of our study is near to many of the other studies in the literature. We were unable to find any difference between the two surgical groups. Therefore, there is little evidence to support the hypothesis that a capsulotomy constructed by femtosecond laser can lead to a more precise effective lens position (ELP).

On the other hand, a Prospective, multicenter, comparative case series showed inferior refractive outcome of FLACS in comparison to conventional surgery [20]. Overall, more than 93% of eyes had a refractive error within 1 D in both groups. This series includes eyes implanted using conventional IOLs as well as toric IOLs. Toric IOLs were significantly more in FLACS cases (47.4% vs. 34.8%; *P* < 0.0001).

A metanalysis of 14,567 Eyes [21]from 15 randomized controlled trials and 22 observational cohort studies showing no statistically significant difference detected between FLACS and conventional surgery regarding UDVA, CDVA, and MAE (weighted mean difference, − 0.02; 95% CI, − 0.07 to 0.04; *P* = 0.57). Analysis of safety parameters revealed that there were no statistically significant differences in the incidence of overall complications between FLACS and conventional surgery; however, posterior capsular tears were significantly more common in FLACS versus CPCS (relative risk, 3.73; 95% CI, 1.50–9.25; *P* = 0.005). The relative small sample number made our study having a small complication rate. Further studies on large scale may be useful to assess various FLACS related complications and their management. FLACS in special situations as in white cataract, Fuchs dystrophy, post radial keratotomy, post keratoplasty, and in congenital cataract will be of a great impact in revealing FLACS potentials.

In this study, the mean SIA was 0.35 ± 0.67 D for the femtosecond laser-constructed corneal wounds and 0.901 ± 0.882 D for the manual keratome (*p* = 0.015). Femtosecond laser is capable of constructing precise multiplanar wounds that may be more secure against post-operative wound leakage. That may make femtosecond laser improve the prediction of corneal shape and the calculation of toric IOLs in turn. To our knowledge, our study is one of the few studies comparing SIA between femtosecond laser and keratome wounds in the literature.

In our study, a statistically significant reduction in residual refractive astigmatism at 6 months postoperatively was found in both groups. A combined phacoemulsification and a single arcuate keratotomy was performed using the VICTUS femtosecond laser platform in a series of 54 eyes of 54 patients who had mean postoperative SIA of 1.20 ± 0.68 D that is higher than our series [22].The mean pre- and post-operative astigmatism was - 1.33 ± 0.57 D and - 0.87 ± 0.56 D respectively which is different from FLACS cases in our series that have a pre- and post-operative astigmatism of - 1.06 ± 1.02 D to − 0.64 ± 0.62 D respectively. Our series had less pre- and post-operative astigmatism that may account for the reduced SIA.

In another series of 48 eyes of 41 patients had cataract surgery (20 FLACS and 28 manual cataract surgery) [23], the mean preoperative corneal astigmatism was 0.81 ± 0.43 D and 0.82 ± 0.52 D for FLACS and conventional surgery groups, respectively. At 3 months after cataract surgery, it was 0.85 ± 0.55 D and 1.03 ± 0.64 D, respectively (*p* > 0.05). The mean SIA in laser and the manual group at 3 months was 0.60 ± 0.73D and 0.37 ± 0.92 D respectively (*p* = 0.318). The average post-operative keratometric astigmatism in the femtosecond group was 0.85 ± 0.55D, compared to manual group 1.03 ± 0.64 at 3 months postoperatively (*p* = 0.332). These finding support well our results of no differences between both groups postoperatively regarding absolute astigmatic error either total clinical or corneal astigmatism.

In our study, both groups showed a statistically significant decrease of total RMS values at 6 months postoperatively compared to preoperative values. There was no statistically difference between both groups postoperatively regarding RMS higher order aberrations. However, RMS total and low order aberrations were statistically significant less in CPCS group when compared to FLACS group 6 months postoperatively. Analysis of selected subtypes of higher order aberrations found postoperatively conventional group had statistically significance better spherical aberrations and trefoil but the difference was of a little clinical significance. The effect of FLACS on ocular aberrations was addressed in many recent studies.

A study of Miháltz et al. [20], compared internal aberrations and quality of vision in eyes treated with the LenSx femtosecond laser and standard manual phacoemulsification. Capsulotomy with the LenSx induced significantly less internal aberrations as measured by the Optical Path Difference (OPD) scanner (NIDEK Inc., Japan). At all measured cycles per degree, the femtosecond treated eyes had lower values of intraocular vertical tilt and coma aberrations.

In Wang et al. [24] study, all wavefront measurements increased significantly at 2 months and 2 years (*P* = 0.007), except spherical aberration (*P* = 0.150) in a series of 50 eyes operated using the Victus platform. There was no significant difference in higher-order aberrations between 2 months and 2 years postoperatively (*P* = 0.486). Corneal astigmatism and higher-order aberration measurements were obtained at the 6-mm zone from corneal topography (Nidek OPD-Scan III; Nidek Technologies). In our series, we measured aberrations in an undilated pupil that mimics the physiological baseline of eyes. Also, lower order aberrations were not assessed in this series in contrast to our study.

An insignificant change of the total HOAs was published by another study [25]. The spherical aberration value is significant and slightly more positive due to the flattening of the cornea due to the incision. It has reported an insignificant difference in Postoperative trefoil after 1 month of the surgery-related changes in corneal wave front aberrations were dependent on incision size. It seems that 2 mm was the limit around which no optical changes are induced by cataract surgery in the human cornea.

Mastropasqua et al. [14] compared functional and morphological outcomes of femtosecond laser clear corneal incision versus manual clear corneal incision during cataract surgery. They stated that there were no statistically significant differences between the two groups regarding corrected distance visual acuity, surgically induced astigmatism, and corneal aberrations. Keratometric astigmatism was significantly lower in the femtosecond laser group at 30 and 180 days postoperatively. Also, femtosecond laser clear corneal incisions showed a better morphology with lower percentage of endothelial and epithelial gaping and endothelial misalignment compared to the manual technique.

Our study has some potential limitations. For new techniques evaluation, randomized control trials (RCT) represents best level of evidence. The reason for lack of randomization in our study is the relatively high cost of FLACS making it only available at private bases on patient demand. The current study is a prospective non-randomized comparative case-control series of FLACS versus conventional cataract surgery. Due to the current cost of FLACS and the lack of necessary research funding and infrastructure for a RCT, this study design was chosen as it represents the next best level of evidence. Furthermore, RCTs are conducted in artificial trial environments with rigid inclusion and exclusion criteria and may not reflect everyday practice. Our post-marketing study could reflect real clinical practice more closely, where clinicians are faced with. Therefore, data from this study have important merits and represent a significant landmark study on refractive outcomes of FLACS.

## Conclusion

In our study, there was a small beneficial effect from the femtosecond laser–assisted capsulotomy and lens fragmentation technique compared with the manual capsulorhexis method. Femtosecond laser–assisted cataract surgery was a safe and precise procedure but enhanced visual outcomes only minimally when compared to conventional cataract surgery in experienced hands. Both FLACS and manual surgeries can achieve a high efficacy, predictability and safety with slight superior outcomes in FLACS.

## Data Availability

Available upon request from the authors.

## Abbreviations

AE: Angle of error
CDVA: Corrected distance visual acuity
CI: Confidence interval
CPCS: Conventional phacoemulsification cataract surgery
ELP: Effective lens position
FLACS: Femtolaser assissted cataract surgery
HOA: High orders aberrations
IOL: Intraocular lens
OPD: Optical path difference
RCT: Randomized controlled study
SIA: Surgically induced astigmatism
UDVA: Uncorrected distance visual acuity
